# A case report of familial type 2 diabetes mellitus combined with hypothyroidism and multiple autoimmune diseases

**DOI:** 10.3389/fendo.2025.1599546

**Published:** 2025-08-04

**Authors:** Xuan Su, Yang Yang, Haotian Jiang, Xiuchang Lai, Yujia Qu

**Affiliations:** ^1^ The First Clinical College, Wuhan University of Science and Technology, Wuhan, China; ^2^ Department of Endocrinology, Tianyou Hospital Affiliated to Wuhan University of Science and Technology, Wuhan, China

**Keywords:** type 2 diabetes, hypothyroidism, Hashimoto’s thyroiditis, autoimmune disease, case report

## Abstract

Gene mutations in the Janus kinase/signal transducer and activator of transcription signaling (JAK/STAT) pathway can promote the occurrence of type 2 diabetes mellitus (T2DM) and autoimmune diseases. We report on two patients with T2DM (a mother and her adult son) with concomitant Hashimoto’s thyroiditis and autoimmune diseases. The son was diagnosed with systemic sclerosis and antiphospholipid syndrome, while the mother was diagnosed with primary biliary cholangitis. Both diagnoses occurred simultaneously. These cases highlight that, in clinical practice, careful symptom assessment, thorough history-taking, standardized physical examination, and obtaining a detailed family history are important. This reduces the misdiagnosis and missed diagnosis rates, enabling early diagnosis and treatment, thereby improving patient outcomes. While genetic testing was not performed in these two patients, this represents a potential direction for future research.

## 1 Introduction

Type 2 diabetes mellitus (T2DM) is a disease caused by a complex interplay of genetic, epigenetic, and environmental factors and is characterized by insulin resistance and insufficient insulin secretion. Research has shown that T2DM clusters in families, and specific causative genes and susceptibility genes associated with the disease have been identified (1). The Janus kinase/signal transducer and activator of transcription (JAK/STAT) signaling pathway is an important intracellular signaling pathway discovered in recent years. Numerous studies have shown that there is dysregulation of the JAK/STAT signaling pathway in T2DM and autoimmune diseases. The combination of T2DM with autoimmune diseases is rarely reported, particularly in the case of T2DM in an immediate family member with multiple autoimmune diseases. These cases suggest that genetic factors may regulate the co-occurrence of T2DM and autoimmune diseases. Detection of the JAK/STAT signaling pathway genes is a direction worthy of further research.

## 2 Case presentation

### 2.1 Case 1

*Presenting complaint*: A 55-year-old man presented on May 10, 2024, with bilateral hand numbness over 1 year, worsened by dry mouth, polydipsia, and polyuria over 4 months. Blurred vision occurred 1 week prior to admission. The outpatient point-of-care capillary blood glucose was 15.2 mmol/L. Urinalysis showed glucose 1+ and protein 1+. He was admitted for management of T2DM and suspected diabetic nephropathy. His mental status, appetite, sleep, bowel habits, physical strength, and body weight were normal.

*Examination*: Vitals: temperature, 36.4°C; pulse, 74/min (regular); blood pressure (BP), 140/89 mmHg; respiratory rate (RR), 20/min (regular). His BMI was 22 kg/m^2^. General: alert, ambulated independently, appeared fatigued, but no icterus. His skin showed multiple hypopigmented macules on the forehead, periorbital areas, mouth corners, neck, and bilateral dorsal hands. Lymphatics: no palpable superficial lymphadenopathy. Cardiopulmonary: auscultation clear. Abdomen: soft, non-tender, no rebound tenderness, and no masses. Sclerodactyly with fingertip swelling was noted in both hands, but no lower limb edema. Dorsalis pedis (DP) pulses were palpable and symmetric bilaterally. Neuromuscular examination revealed normal limb muscle strength and tone.

*Investigations*: glycosylated hemoglobin (HbA1c), 14.5% ↑; free triiodothyronine (FT3), 1.15 pg/ml ↓; free thyroxine (FT4), <3.00 pg/ml ↓; thyrotropin (thyroid-stimulating hormone, TSH), >100.000 mU/L ↑; anti-thyroglobulin antibody, 182.41 IU/ml (+); and anti-U1-snRNP antibody (+), anti-PM-Scl antibody (+), anti-Jo-1 antibody (±), anti-Ku antibody (±), and anti-cardiolipin anti-IgM (+). Other investigation results are shown in **Table 1**. Imaging: thyroid ultrasound (with cervical lymph nodes), solid hypoechoic nodule in the right lobe of the thyroid gland of TI-RADS (Thyroid Imaging Reporting and Data System) grade III, and bilateral cervical lymph nodes visible. Screening for diabetic complications: no abnormal changes found on fundus photography; the sensory examination suggests that vibration, touch and temperature sensation are normal. Doppler flowmetry of the extremities: right lower extremity posterior tibial artery (PT) ankle–branchial index (ABI) of 1.10 and DP ABI of 1.10; left lower extremity PT ABI of 1.19 and DP ABI of 1.19.

**Table 1:** Comparison of the results during hospitalization and the follow-up investigations.

Test	05/10/2024	06/03/2024 (after 1 month)	07/08/2024 (after 2 months)	09/02/2024 (after 4 months)	11/02/2024 (after 6 months)	12/02/2024 (after 7 months)	01/06/2025 (after 8 months)
Thyrotropin (mU/L)	>100.00↑	101.00↑	7.88↑	–	–	5.29↑	6.98↑
Free triiodothyronine (pg/ml)	1.15↓	2.19↓	3.58	–	–	2.70	2.70
Free thyroxine (pg/ml)	<3.00↓	3.61↓	8.95	–	–	6.56	9.26
Alanine aminotransferase (U/L)	52.30↑	43.10	39.40	21.90	–	–	–
Aspartate aminotransferase (U/L)	81.30↑	48.10↑	24.10	19.80	23.60	31.30	–
Gamma glutamyl aminotransferase (U/L)	31.90	85.30↑	47.40	33.50	–	–	–
Anti-U1-SnRNP antibody	+	–	–	±	–	–	–
Anti-PM-Scl antibody	+	–	–	±	–	–	–
Anti-JO-1 antibody	±	–	–	-	–	–	–
Anti-Ku antibody	±	–	–	-	–	–	–
Anti-cardiolipin antibody IgM	±	–	–	–	–	–	–
Oral glucose tolerance test (mmol/L)	Fasting 18.6↑	–	–	–	–	–	–
	60 min 27.4↑	–	–	–	–	–	–
	120 min 32.6↑	–	–	–	–	–	–
	180 min 35.1↑	–	–	–	–	–	–
C-peptide release test (ng/ml)	Fasting 1.67	–	–	–	–	–	–
	60 min 2.2↓	–	–	–	–	–	–
	120 min 2.92↓	–	–	–	–	–	–
	180 min 3.07	–	–	–	–	–	–
Insulin release test (μIU/ml)	Fasting 2.40	–	–	–	–	–	–
	60 min 5.40↓	–	–	–	–	–	–
	120 min 7.70	–	–	–	–	–	–
	180 min 6.90	–	–	–	–	–	–

The patient’s thyroid function and liver function gradually returned to normal. The antibody reactivity of the anti-U1-snRNP and anti-PM-Scl antibodies shifted from positive to weakly positive, while that of the anti-Jo-1 and anti-Ku antibodies shifted from weakly positive to negative.

*Diagnoses*: T2DM, type 2 diabetic nephropathy (stage IIIB), type 2 diabetic peripheral neuropathy, hypothyroidism (Hashimoto’s thyroiditis, HT), overlap syndrome [connective tissue disease: systemic sclerosis (SSc) and antiphospholipid syndrome], vitiligo, thyroid nodule, and liver dysfunction.

*Treatments*: 1) Type 2 diabetes: mixed protamine zinc recombinant human insulin lispro injection (50 R), 26 IU, subcutaneous injection, twice a day; acarbose capsule, two capsules, oral before food. 2) Liver dysfunction: bicyclol tablets, one tablet, three times/day. 3) Type 2 diabetic nephropathy: BaiLingJiaoNang, four capsules, three times/day. 4) Hypothyroidism: levothyroxine sodium tablets, 50 μg, once a day. 5) Autoimmune diseases: total glucosides of White Peony capsules, two capsules, twice a day; hydroxychloroquine sulfate tablets, one tablet, twice a day; prednisone, 10 mg, twice a day. 6) Peripheral neuropathy: vitamin B1 tablets, one tablet, once a day; mecobalamin tablets, one tablet, once a day. 7) Prevention of osteoporosis: calcitriol capsules, one capsule, once a day; calcium carbonate and vitamin D3 tablets, one tablet, once a day.

**Figure 1:**
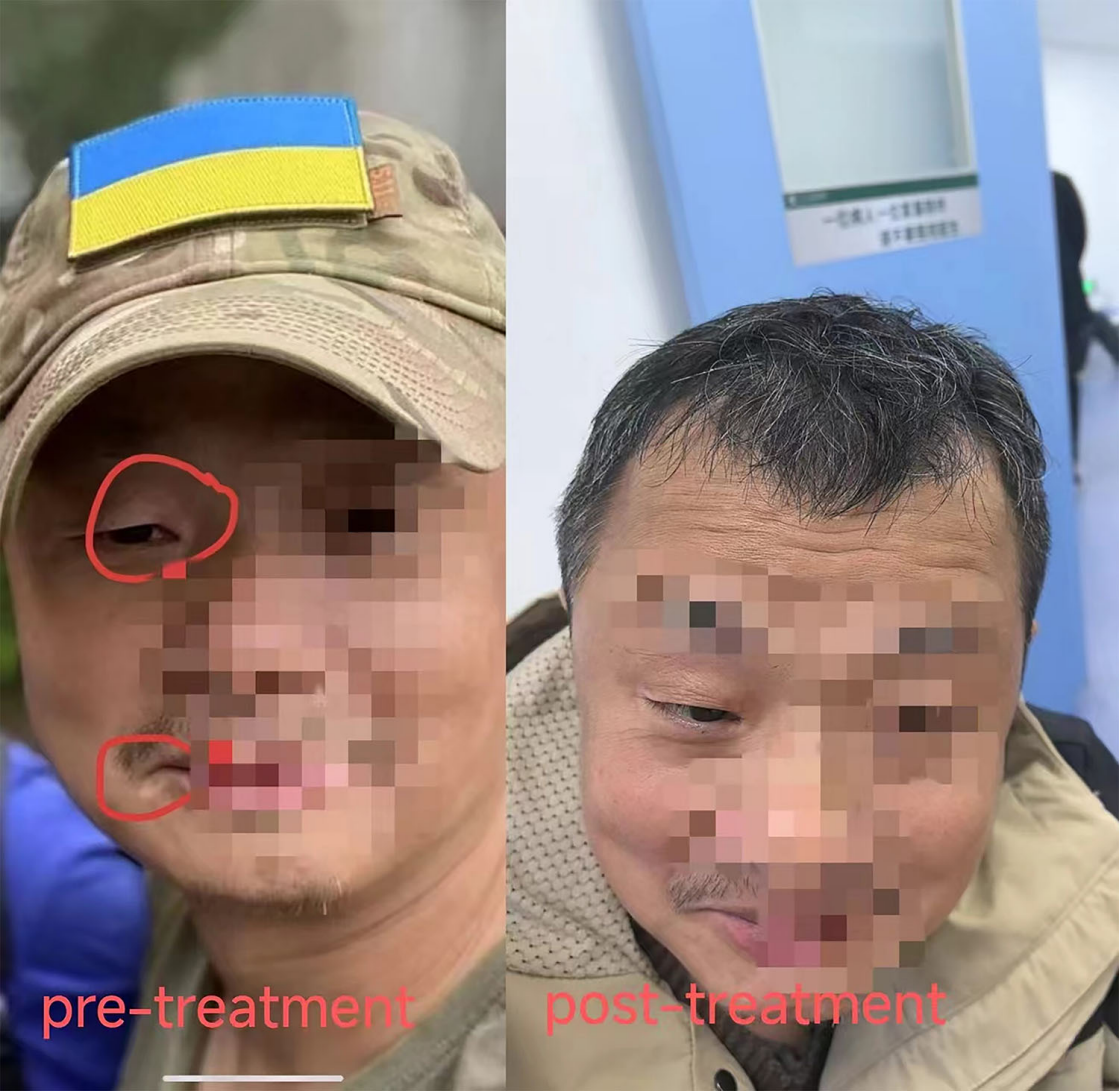
Case 1 before and after treatment.

Follow-up: Regular follow-up visits were conducted after discharge. The multiple hyperpigmented macules on the patient’s skin resolved completely (**Figure 1**). The patient’s thyroid function and liver function gradually returned to normal. The antibody reactivity of the anti-U1-snRNP and anti-PM-Scl antibodies shifted from positive to weakly positive, while that of the anti-Jo-1 and anti-Ku antibodies shifted from weakly positive to negative (**Table 1**).

### 2.2 Case 2

**Figure 2:**
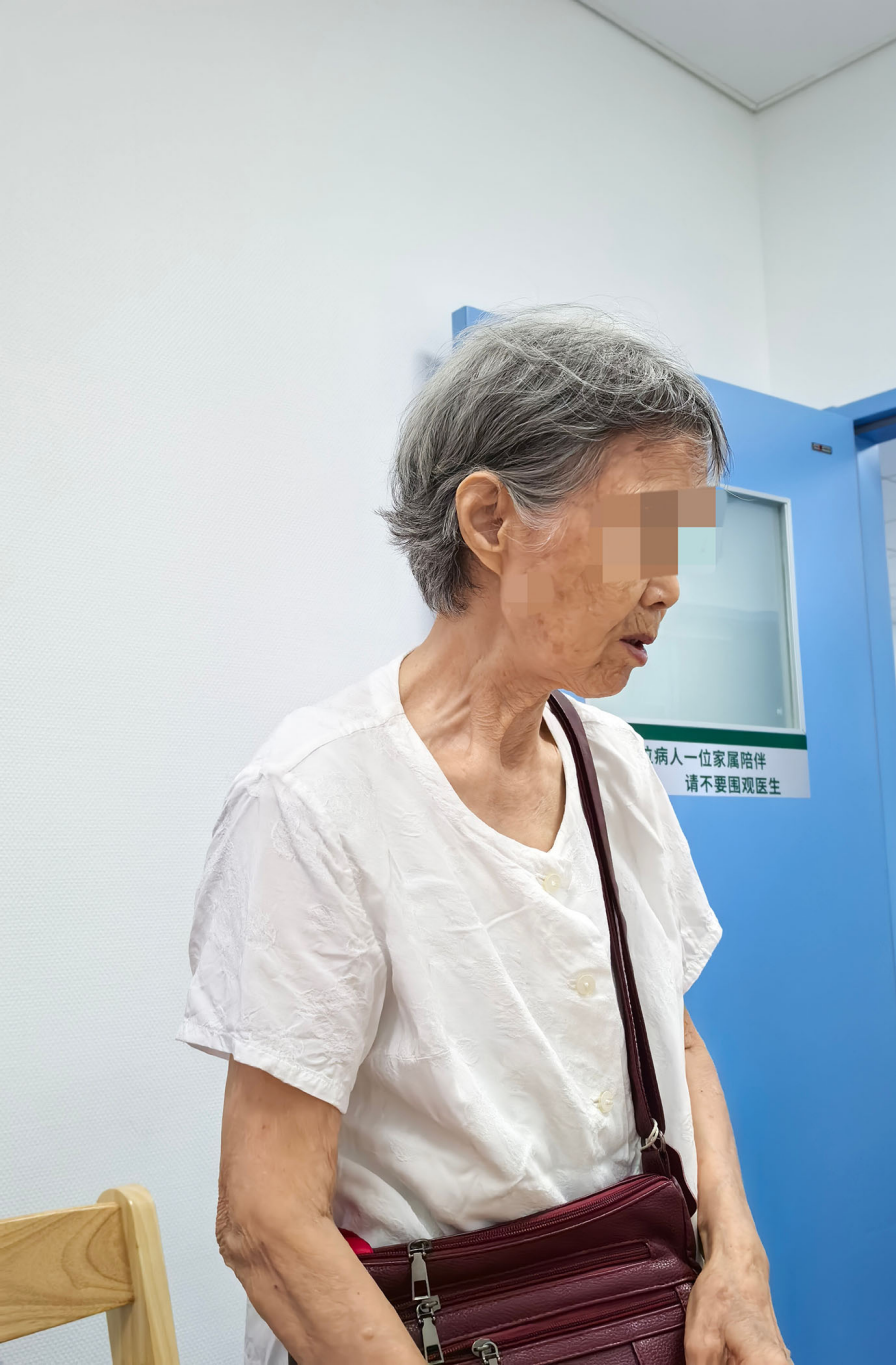
The photograph of case 2.

*Presenting complaint*: An 81-year-old woman (**Figure 2**) presented on May 20, 2024, with 6 months of hyperglycemia and 1 month of intermittent fatigue. During the course of the disease, the patient gradually developed bilateral blurred vision, foamy urine, heaviness in the bilateral lower limbs, dry mouth, polydipsia, and intermittent numbness of limbs. She was admitted for management of T2DM. Since the disease onset, the patient’s mental status, appetite, and sleep have been poor, but her bowel habits were normal. She developed a significantly reduced physical endurance and a 6-kg unintentional weight loss over the past year.

*Examination*: Vitals: temperature, 35.9°C; pulse, 74/min (regular); RR, 20/min (regular); BP, 132/74 mmHg; BMI, 16 kg/m^2^. General: alert, ambulated independently, appeared fatigued, and no icterus. Lymphatics: no palpable superficial lymphadenopathy. Cardiopulmonary: auscultation clear. Abdomen: soft, non-tender, no rebound tenderness, no masses, no right lower extremity edema, but mild edema over the left foot dorsum. Neuromuscular: normal limb muscle strength and tone.

*Investigations*: HbA1c, 6.2% ↑; β-hydroxybutyric acid, 0.49 mmol/L ↑; TSH, 5.149 mU/L ↑; FT3, 3.21 pg/ml; FT4, 8.5 pg/ml; anti-thyroglobulin antibody, 342.50 IU/ml (+); and anti-AMA-M2 antibody (+), anti-mitochondrial antibody type II (+), anti-smooth muscle antibodies (+), and antinuclear antibody IgG (cytoplasmic mitochondrial pattern, 1:160 titer). Other investigation results are shown in **Table 2**. Imaging: dual-energy X-ray bone mineral density (BMD) *T*-value, less than −2.5. Thyroid ultrasound: increased thyroid blood flow signal and small solid nodule in the left lobe of the thyroid gland, TI-RADS grade II. Diabetes mellitus specialist examination: in both eyes, the fundus image did not show abnormal changes; the sensory examination suggests that vibration, touch and temperature sensation are normal, and cool temperature sensation is normal. Doppler flowmetry of the extremities suggests left ABIs of 0.93 and 0.93 (for PT and DP, respectively) and right ABIs of 0.93 and 1.08 (for PT and DP, respectively).

**Table 2:** Comparison of the results during hospitalization and the follow-up investigations.

Test	05/20/2024	06/24/2024 (after 1 month)	07/29/2024 (after 2 months)	08/28/2024 (after 3 months)	10/21/2024 (after 5 months)	11/25/2024 (after 6 months)
Thyrotropin (mU/L)	5.15↑	5.29↑	–	4.64	–	3.86
Free triiodothyronine (pg/ml)	3.21	3.05	–	2.85	–	3.06
Free thyroxine (pg/ml)	8.50	9.14	–	9.28	–	9.53
Alanine aminotransferase (U/L)	48.10↑	14.40	34.30	16.10	18.60	35.00
Aspartate aminotransferase (U/L)	38.70↑	26.00	36.90↑	27.30	26.70	33.70
Gamma glutamyl aminotransferase (U/L)	77.60↑	49.50↑	50.50↑	37.40	30.90	41.70
Oral glucose tolerance test (mmol/L)	Fasting 4.6	–	–	–	–	–
	60 min 12.57↑	–	–	–	–	–
	120 min 17.32↑	–	–	–	–	–
	180 min 13.96↑	–	–	–	–	–
C-peptide release test (ng/ml)	Fasting 1.43	–	–	–	–	–
	60 min 7.57	–	–	–	–	–
	120 min 9.66	–	–	–	–	–
	180 min 10.24↑	–	–	–	–	–
Insulin release test (μIU/ml)	Fasting 4.20	–	–	–	–	–
	60 min 32.80	–	–	–	–	–
	120 min 38.90↑	–	–	–	–	–
	180 min 28.10 ↑	–	–	–	–	–
Anti-smooth muscle antibody	+	–	–	–	–	–
Antinuclear antibody IgG	Cytoplasmic mitochondrial pattern, 1:160 titer	–	–	–	–	–
Anti-mitochondrial antibody type II	+	–	–	–	–	–
Anti-AMA-M2 antibody	+	–	–	–	–	–

The patient’s thyroid function and liver function gradually returned to normal.

*Diagnoses*: T2DM, type 2 diabetic peripheral neuropathy, type 2 diabetic ketosis, type 2 diabetic nephropathy, autoimmune liver disease (primary biliary cholangitis, PBC), HT, thyroid nodule, senile osteoporosis, thrombocytopenia, and liver dysfunction.

*Treatments*: 1) Type 2 diabetes: alogliptin benzoate tablets, 12.5 mg, taken orally before breakfast. 2) Autoimmune liver disease (PBC): ursodeoxycholic acid capsules, two capsules, twice a day. 3) Hypothyroidism: levothyroxine sodium tablets, 1/4 tablet, every other day for treatment. 4) Osteoporosis: calcitriol capsules, one capsule, once a day; calcium carbonate and vitamin D3 tablets, one tablet, once a day; desucumab injection, 60 mg, semi-annual treatment. 5) Peripheral neuropathy: mecobalamin tablets, one tablet, once a day. 6) Thrombocytopenia: caffeic acid tablets, three times/day. 7) Liver dysfunction: HuGanKeLi, one sachet, three times/day; bicyclol tablets, one tablet, three times/day.

*Follow-up*: Regular follow-up visits were conducted after discharge. The patient’s thyroid function and liver function gradually returned to normal. The follow-up results are shown in **Table 2**.

## 3 Discussion

The final diagnoses of case 1 were T2DM, HT, and connective tissue disease (SSc and antiphospholipid syndrome). The final diagnoses of case 2 were T2DM, HT, and autoimmune liver disease (PBC). Although both patients had preserved β-cell function and negative diabetes-associated autoantibodies, they do not meet the diagnostic criteria for latent autoimmune diabetes in adults (LADA) (2). It is necessary to be vigilant of the possibility of LADA. Therefore, ruling out LADA requires longitudinal monitoring. During follow-up, long-term monitoring of C-peptide and diabetes-associated autoantibodies in these patients is required.

The conditions of the two patients in this report might be related to the JAK/STAT signaling pathway. This pathway has three main components: cytokine receptors, Janus kinases (JAKs), and signal transducers and activators of transcription (STATs) (3). JAKs are non-receptor tyrosine kinases composed of four members: JAK1, JAK2, JAK3, and TYK2. Dimerized kinases bind cytokine receptors, phosphorylating the intracellular tyrosine residues to propagate activation signals. Of these, JAK1, JAK2, and TYK2 are ubiquitously expressed, whereas the expression of JAK3 is restricted to the bone marrow and the lymphatic system (4). The human STAT family consists of seven members: STAT1, STAT2, STAT3, STAT4, STAT5a/b, and STAT6 (5).

Gene mutations in the JAK/STAT signaling pathway can drive the release of interferons (IFNs) and other cytokines. These IFNs and other cytokines can further enhance the activation of the JAK/STAT signaling pathway, establishing a pathogenic positive feedback loop that promotes multi-organ damage. IFNs are cytokines that can be classified into three groups: type I (IFN-α, IFN-β, IFN-δ, IFN-ϵ, IFN-κ, IFN-τ, IFN-ω, and IFN-ζ), type II (IFN-λ), and type III ( IFN-λ) (6). After transcriptional activation and mRNA translation, type I IFNs (IFN-I) are secreted by immune cells to adjacent cells and bind to two receptor subunits: IFN-α receptor 1 (IFNAR1) and IFNAR2. These two receptors are respectively associated with TYK2 and JAK1 (7). The dimerization of the receptor initiates the autophosphorylation of JAK1. JAK1 subsequently phosphorylates and activates the STAT1 and STAT2 proteins. These proteins form a complex with interferon regulatory factor 9 (IRF9), eventually forming a well-characterized complex, IFN-stimulated gene factor 3 (ISGF3). ISGF3 translocates into the nucleus and binds to the IFN-stimulated response elements (ISREs) in the promoters of genes, thereby promoting the transcription of IFN-stimulated genes (ISGs) (8). JAKs also mediate the signaling pathways of various other cytokines, including interleukin-2 (IL-2), IL-4, IL-6, IL-7, IL-9, IL-10, IL-12, IL-13, IL-15, and IL-21 (9).

Numerous studies have shown that mutations in the STAT-related genes can lead to the occurrence of autoimmune diseases. For instance, gain-of-function (GOF) mutations in *STAT1* amplify IFN-α/β signaling, predisposing to disorders ranging from autoimmune thyroiditis to systemic lupus erythematosus (10, 11). In thyroiditis, infiltrating pro-inflammatory cytokines (e.g., TNF-α and IFN-γ) induce inflammasome activation and thyrocyte apoptosis (12). Similarly, the STAT3 P471R variant hyperactivates the T helper 17 (Th17) pathway, increasing IL-17 production and triggering autoimmunity (13). Conversely, STAT5B deficiency impairs regulatory T-cell function, facilitating multi-organ autoimmune manifestations such as eczema, juvenile idiopathic arthritis, and immune thrombocytopenia (14). A meta-analysis further implicates the STAT4 rs7574865 T-allele in the increased risk of SSc and antiphospholipid syndrome (15).

The onset of SSc is associated with the abnormal increase of cytokines. An elevated IL-4 is a key mediator. It synergizes with IL-13 and transforming growth factor beta (TGF-β) to amplify the inflammatory and pro-fibrotic responses, facilitating the pathogenic T cell-fibroblast crosstalk (16, 17). Phosphorylated STAT1/3 levels increase in peripheral blood T cells and monocytes, and cells with STAT3 phosphorylation are also found in skin sections of patients with SSc (18). Such IL-4-driven signaling promotes fibrosis via JAK/STAT transcriptional programs. The level of IFN-I are also associated with severe cutaneous, pulmonary, and musculoskeletal involvement in patients with SSc (19). Critically, the anti-IFNAR monoclonal antibody anifrolumab suppresses disease activity (20), confirming the central role of IFN-I in SSc.

IFN-γ plays a central role in the pathogenesis of vitiligo. IFN-γ acts on keratinocytes to induce the secretion of the serum chemokines CXC ligand 9 (CXCL9) and CXCL10. These chemokines recruit the CXC receptor 3 (CXCR3) CD8^+^ T cells. The activated CXCR3 CD8^+^ T cells can induce melanocyte apoptosis and IFN-γ secretion (21).

IFN-I promote liver injury in both metabolic and cholestatic contexts. In fatty liver diseases, they amplify Toll-like receptor 4 signaling in macrophages and recruit CD8^+^ T cells (22, 23). In PBC, portal tract immune cells overexpress IFN-I (24), indicating a common effector mechanism.

The involvement of the JAK/STAT signaling pathway in the pathogenesis of T2DM is increasingly recognized. Genetic variants in JAK2 exemplify this link: the allele/genotype frequencies of rs10974914 and rs10815157 differ significantly between diabetic and control cohorts, with the rs10974914-AA genotype increasing the T2DM risk while the rs10815157-C allele conferring protection (25). Mechanistically, JAK2 overexpression amplifies the insulin promoter activity (26), whereas phosphorylated STAT3 impairs the insulin signaling and glucose uptake in skeletal muscle, driving insulin resistance (27). Complementary evidence implicates STAT4 polymorphisms (e.g., rs7574865) in T2DM susceptibility among Chinese Han populations (28).

## 4 Conclusion

These cases underscore that, in clinical diagnosis and treatment, it is important to closely observe the symptoms and signs of patients with familial T2DM and to pay attention to the autoimmune manifestations for early diagnosis and treatment. The association of T2DM and autoimmune diseases with the JAK/STAT signaling pathway has been well studied, and patients with T2DM combined with autoimmune diseases may have specific JAK/STAT variants. The characteristics of these cases suggest a possible genetic predisposition, and genetic testing of these patients could help identify potential genetic risks. Neither patient underwent genetic testing. Future research could investigate the association of specific JAK/STAT variants with T2DM complicated with autoimmune diseases through targeted genetic screening.

## Data Availability

The original contributions presented in the study are included in the article/**Supplementary Material**. Further inquiries can be directed to the corresponding author.

## References

[1] Zhu Y Wang W Li Q. Research progress on genetic predisposition and associated genes of type 2 diabetes. Chin J Prev Contr Chron Dis. (2015) 23(3):229–32.

[2] Qiu J Xiao Z Zhang Z Luo S Zhou Z. Latent autoimmune diabetes in adults in China. Front Immunol. (2022) 13:977413. doi: 10.3389/fimmu.2022.977413, PMID: 36090989 PMC9454334

[3] Wang Y Leng J. The relationship between JAK-STAT signaling pathway and painful diabetic peripheral neuropathy. Chin J Diffic and Compl Cas. (2018) 17:1176–8.

[4] Gao Q Liang X Shaikh AS Zang J Xu W Zhang Y. JAK/STAT signal transduction: promising attractive targets for immune, inflammatory and hematopoietic diseases. Curr Drug Targets. (2018) 19:487–500. doi: 10.2174/1389450117666161207163054, PMID: 27928945

[5] Seif F Khoshmirsafa M Aazami H Mohsenzadegan M Sedighi G Bahar M. The role of JAK-STAT signaling pathway and its regulators in the fate of T helper cells. Cell communication Signaling. (2017) 15:1–3. doi: 10.1186/s12964-017-0177-y, PMID: 28637459 PMC5480189

[6] Li S Gong M Zhao F Shao J Xie Y Zhang Y. Type I interferons: Distinct biological activities and current applications for viral infection. Cell Physiol Biochem. (2018) 51:2377–96. doi: 10.1159/000495897, PMID: 30537741

[7] Platanias LC. Mechanisms of type-i- and type-II-interferon-mediated signalling. Nat Rev Immunol. (2005) 5:375–86. doi: 10.1038/nri1604, PMID: 15864272

[8] Lukhele S Boukhaled GM Brooks DG. Type I interferon signaling, regulation and gene stimulation in chronic virus infection. Semin Immunol. (2019) 43:101277. doi: 10.1016/j.smim.2019.05.001, PMID: 31155227 PMC8029807

[9] Stark GR Cheon H Wang Y. Responses to cytokines and interferons that depend upon jaks and stats. Cold Spring Harbor Perspect Biol. (2017) 10. doi: 10.1101/cshperspect.a028555, PMID: 28620095 PMC5749152

[10] Boisson-Dupuis S Kong XF Okada S Cypowyj S Puel A Abel L. Inborn errors of human STAT1: allelic heterogeneity governs the diversity of immunological and infectious phenotypes. Curr Opin Immunol. (2012) 24:364–78. doi: 10.1016/j.coi.2012.04.011, PMID: 22651901 PMC3477860

[11] Toubiana J Okada S Hiller J Oleastro M Lagos Gomez M Aldave Becerra JC. Heterozygous STAT1 gain-of-function mutations underlie an unexpectedly broad clinical phenotype. Blood J Am Soc Hematol. (2016) 127:3154–64. doi: 10.1182/blood-2015-11-679902, PMID: 27114460 PMC4920021

[12] Guo Q Wu Y Hou Y Liu Y Liu T Zhang H. Cytokine secretion and pyroptosis of thyroid follicular cells mediated by enhanced NLRP3, NLRP1, NLRC4, and AIM2 inflammasomes are associated with autoimmune thyroiditis. Front Immunol. (2018) 9:1197. doi: 10.3389/fimmu.2018.01197, PMID: 29915579 PMC5994487

[13] Wienke J Janssen W Scholman R Spits H van Gijn M Boes M. A novel human STAT3 mutation presents with autoimmunity involving Th17 hyperactivation. Oncotarget. (2015) 6:20037. doi: 10.18632/oncotarget.5042, PMID: 26343524 PMC4652985

[14] Kanai T Jenks J Nadeau KC. The STAT5b pathway defect and autoimmunity. Front Immunol. (2012) 3:234. doi: 10.3389/fimmu.2012.00234, PMID: 22912632 PMC3418548

[15] Liang YL Wu H Shen X Li PQ Yang XQ Liang L. Association of STAT4 rs7574865 polymorphism with autoimmune diseases: a meta-analysis. Mol Biol Rep. (2012) 39:8873–82. doi: 10.1007/s11033-012-1754-1, PMID: 22714917

[16] Gasparini G Cozzani E Parodi A. Interleukin-4 and interleukin-13 as possible therapeutic targets in systemic sclerosis. Cytokine. (2020) 125:154799. doi: 10.1016/j.cyto.2019.154799, PMID: 31400638

[17] Higashioka K Kikushige Y Ayano M Kimoto Y Mitoma H Kikukawa M. Generation of a novel CD30+ B cell subset producing GM-CSF and its possible link to the pathogenesis of systemic sclerosis. Clin Exp Immunol. (2020) 201:233–43. doi: 10.1111/cei.13477, PMID: 32538493 PMC7419935

[18] Kitanaga Y Imamura E Nakahara Y Fukahori H Fujii Y Kubo S. *In vitro* pharmacological effects of peficitinib on lymphocyte activation: a potential treatment for systemic sclerosis with JAK inhibitors. Rheumatology. (2020) 59:1957–68. doi: 10.1093/rheumatology/kez526, PMID: 31764973 PMC7382595

[19] Liu X Mayes MD Tan FK Wu M Reveille JD Harper BE. Correlation of interferon-inducible chemokine plasma levels with disease severity in systemic sclerosis. Arthritis Rheumatism. (2012) 65:226–35. doi: 10.1002/art.37742, PMID: 23055137 PMC3687352

[20] Guo X Higgs BW Bay-Jensen AC Karsdal MA Yao Y Roskos LK. Suppression of T cell activation and collagen accumulation by an anti-IFNAR1 MAB, anifrolumab, in adult patients with systemic sclerosis. J Invest Dermatol. (2015) 135:2402–9. doi: 10.1038/jid.2015.188, PMID: 25993119

[21] Zhao Y Lu N Wang X Chen W Zhang L. Exploration of the immunological pathogenesis of vitiligo. Chin J Dermato Venerol Integ Trad W Med. (2022) 21:381–3.

[22] Zhai Y Qiao B Gao F Shen X Vardanian A Busuttil RW. Type I, but not type II, interferon is critical in liver injury induced after ischemia and reperfusion. Hepatology. (2008) 47:199–206. doi: 10.1002/hep.21970, PMID: 17935177

[23] Ghazarian M Revelo XS Nøhr MK Luck H Zeng K Lei H. Type I interferon responses drive intrahepatic T cells to promote metabolic syndrome. Sci Immunol. (2017) 2. doi: 10.1126/sciimmunol.aai7616, PMID: 28567448 PMC5447456

[24] Bae HR Hodge DL Yang G Leung PSC Chodisetti SB Valencia JC. The interplay of type I and type II interferons in murine autoimmune cholangitis as a basis for sex-biased autoimmunity. Hepatology. (2018) 67:1408–19. doi: 10.1002/hep.29524, PMID: 28921595 PMC5856578

[25] Zhang Y Lin C Chen R Luo L Huang J Liu H. Association analysis of SOCS3, JAK2 and STAT3 gene polymorphisms and genetic susceptibility to type 2 diabetes mellitus in Chinese population. Diabetol Metab Syndrome. (2022) 14:4. doi: 10.1186/s13098-021-00774-w, PMID: 34991691 PMC8734348

[26] Zhang Y Zhou B Deng B Zhang F Wu J Wang Y. Amyloid-β induces hepatic insulin resistance *in vivo* via JAK2. Diabetes. (2013) 62:1159–66. doi: 10.2337/db12-0670, PMID: 23223021 PMC3609589

[27] Mashili F Chibalin AV Krook A Zierath JR. Constitutive STAT3 phosphorylation contributes to skeletal muscle insulin resistance in type 2 diabetes. Diabetes. (2013) 62:457–65. doi: 10.2337/db12-0337, PMID: 23043161 PMC3554355

[28] Cui J Tong R Xu J Tian Y Pan J Wang N. Association between STAT4 gene polymorphism and type 2 diabetes risk in Chinese Han population. BMC Med Genomics. (2021) 14:1–6. doi: 10.1186/s12920-021-01000-2, PMID: 34176465 PMC8237503

