# Supplementary material for: A case report of familial type 2 diabetes mellitus combined with hypothyroidism and multiple autoimmune diseases
Source: Front Endocrinol (Lausanne). 2025 Aug 4;16:1599546. doi: 10.3389/fendo.2025.1599546 (PMC12358270; doi:10.3389/fendo.2025.1599546)
Supplement: Supplementary file 1 [file DataSheet1.pdf]

Supplementary Table 1

Test Name	05/10/2024	06/03/2024(After 1 month)	07/08/2024（After 2 month）	09/02/2024（After 4 month）	11/02/2024（After 6 month）	12/02/2024（After 7 month）	01/06/2025（After 8 month）
White blood cell(*10^9/L)	5.09	\	\	\	\	\	\
Red blood cell(*10^12/L)	4.26↓	\	\	\	\	\	\
Hemoglobin(g/L)	134.00	\	\	\	\	\	\
Blood platelet(*10^9/L)	110.00↓	\	\	\	\	\	\
Creatinine(umol/L)	166.90↑	137.90↑	116.50↑	124.20↑	126.80↑	131.10↑	125.00↑
Uric acid(umol/L)	368.30	401.80	375.90	553.00↑	516.00↑	273.00	267.10
Estimation of glomerular filtration rate[ml/(min/1.73m-2)]	39.12↓	\	60.42↓	55.92↓	54.54↓	52.38↓	55.49↓
Parathyroid hormone(pg/mL)	9.40↓	\	\	15.60	\	\	21.30
Immunoglobulin G4	-	\	\	\	\	\	\
Erythrocyte sedimentation rate(mm/h)	23.4↑	\	\	\	\	\	\
Fibrinogen(g/L)	1.94↓	\	\	\	\	\	\
Potassium(mmol/L)	3.6	\	\	\	\	\	\
Magnesium(mmol/L)	8.03	\	\	\	\	\	\
Calcium(mmol/L)	2.48	\	\	\	\	\	\
Phosphorus(mmol/L)	1.24	\	\	\	\	\	\
Total 25-hydroxyvitamin D(mmol/L)	23.42↓	\	\	\	\	\	\
Glycosylated hemoglobin(%)	14.5 ↑	\	\	\	\	\	\
Urinary microalbumin(mg/L)	62.80 ↑	\	\	\	\	\	\
Urinary creatinine	38.34 ↑	\	\	\	\	\	\
Anti-insulin antibody	-	\	\	\	\	\	\

Anti-islet cell antibody	-	\	\	\	\	\	\
Anti-glutamic acid decarboxylase antibody	-	\	\	\	\	\	\
Anti-tyrosine phosphatase-like protein antibody	-	\	\	\	\	\	\
Anti-smooth muscle antibody	-	\	\	\	\	\	\
Anti-nuclear antibody IgG	-	\	\	\	\	\	\
Anti-soluble liver antigen/hepato-pancreatic antibody	-	\	\	\	\	\	\
Gp210	-	\	\	\	\	\	\
Anti-hepatic and renal microsomal Antibody	-	\	\	\	\	\	\
Anti-mitochondrial antibody type II	-	\	\	\	\	\	\
Anti-hepatocyte solute antigen type I antibody	-	\	\	\	\	\	\
Sp100	-	\	\	\	\	\	\
Hepatitis B virus	-	\	\	\	\	\	\
Hepatitis C virus	-	\	\	\	\	\	\
Antithyroid peroxidase antibody	-	\	\	\	\	\	\
Anti-dsDNA antibody	-	\	\	-	\	\	\
Anti-Nucleosome antibody	-	\	\	-	\	\	\
Anti-Histone Antibody	-	\	\	-	\	\	\
Anti-SmD1 antibody	-	\	\	-	\	\	\
Anti-PCNA antibody	-	\	\	-	\	\	\
Anti-Rib-PO antibody	-	\	\	-	\	\	\
Anti-SS-A/Ro 60 kD antibody	-	\	\	-	\	\	\
Anti-SS-A/Ro 52 kD antibody	-	\	\	-	\	\	\
Anti-SS-B/La antibody	-	\	\	-	\	\	\

Anti-CENP antibody	-	\	\	-	\	\	\
Anti-Scl70 antibody	-	\	\	-	\	\	\
Anti-AMA-M2 antibody	-	\	\	-	\	\	\
Anti-Mi -2 antibody	-	\	\	-	\	\	\
Anti-cardiolipin anti-IgG	-	\	\	\	\	\	\
Anti-cardiolipin anti-IgA	-	\	\	\	\	\	\

Table 1 Comparison of other investigations results during hospitalization and follow-up.

Supplementary Table 2

Test Name	05/20/2024	06/24/2024(After 1 month)	07/29/2024(After 2 month)	08/28/2024(After 3 month)	10/21/2024(After 5 month)	11/25/2024(After 6 month)
White blood cell(*10^9/L)	6.85	8.45	9.35	8.76	\	\
Red blood cell(*10^12/L)	2.95↓	3.32↓	3.24↓	3.18↓	\	\
Hemoglobin(g/L)	99.00↓	115.00	113.00↓	113.00↓	\	\
Blood platelet(*10^9/L)	117.00↓	98.00↓	114.00↓	120.00↓	\	\
Creatinine(μmol/L)	81.00	85.60↑	83.20↑	82.50↑	84.80↑	84.20↑
Uric acid(μmol/L)	265.90	321.20	168.60	222.00	193.90	311.90
Estimation of glomerular filtration rate[ml/(min/1.73m-2)]	\	55.06↓	56.99↓	57.58↓	55.69↓	56.17↓
Erythrocyte sedimentation rate(mm/h)	26.9↑	\	\	\	\	\
Urinary microalbumin(mg/L)	20.50↑	\	\	\	\	\
Urinary creatinine	4.95	\	\	\	\	\
Anti-insulin antibody	-	\	\	\	\	\
Anti-islet cell antibody	-	\	\	\	\	\
Anti-glutamic acid decarboxylase antibody	-	\	\	\	\	\

Anti-tyrosine phosphatase-like protein antibody	-	\	\	\	\	\
Anti-soluble liver antigen/hepato-pancreatic antibody	-	\	\	\	\	\
Gp210	-	\	\	\	\	\
Anti-hepatic and renal microsomal Antibody	-	\	\	\	\	\
Anti-hepatocyte solute antigen type I antibody	-	\	\	\	\	\
Sp100	-	\	\	\	\	\
Antithyroid peroxidase antibody	-	\	\	\	\	\
Anti-dsDNA antibody	-	\	\	\	\	\
Anti-Nucleosome antibody	-	\	\	\	\	\
Anti-Histone Antibody	-	\	\	\	\	\
Anti-SmD1 antibody	-	\	\	\	\	\
Anti-PCNA antibody	-	\	\	\	\	\
Anti-Rib-PO antibody	-	\	\	\	\	\
Anti-SS-A/Ro 60 kD antibody	-	\	\	\	\	\
Anti-SS-A/Ro 52 kD antibody	-	\	\	\	\	\
Anti-SS-B/La antibody	-	\	\	\	\	\
Anti-CENP antibody	-	\	\	\	\	\
Anti-Scl70 antibody	-	\	\	\	\	\
Anti-Mi -2 antibody	-	\	\	\	\	\
Anti-U1-SnRNP antibody	-	\	\	\	\	\
Anti-PM-Scl antibody	-	\	\	\	\	\
Anti-JO-1 antibody	-	\	\	\	\	\
Anti-Ku Antibody	-	\	\	\	\	\

Table 2 Comparison of other investigations results during hospitalization and follow-up.
